# Glycation in Demetalated Superoxide Dismutase 1 Prevents Amyloid Aggregation and Produces Cytotoxic Ages Adducts

**DOI:** 10.3389/fmolb.2016.00055

**Published:** 2016-09-16

**Authors:** Ivana Sirangelo, Filomena M. Vella, Gaetano Irace, Giuseppe Manco, Clara Iannuzzi

**Affiliations:** ^1^Department of Biochemistry, Biophysics and General Pathology, Second University of NaplesNaples, Italy; ^2^Institute of Agro-environmental and Forest Biology, Italian National Research CouncilNaples, Italy; ^3^Institute of Protein Biochemistry, Italian National Research CouncilNaples, Italy

**Keywords:** fALS, SOD1, amyloid aggregation, protein glycation, ages, cytotoxicity

## Abstract

Superoxide dismutase 1 (SOD1) has been implicated with familial amyotrophic lateral sclerosis (fALS) through accumulation of protein amyloid aggregates in motor neurons of patients. Amyloid aggregates and protein inclusions are a common pathological feature of many neurological disorders in which protein aggregation seems to be directly related to neurotoxicity. Although, extensive studies performed on the aggregation process of several amyloidogenic proteins *in vitro* allowed the identification of many physiological factors involved, the molecular mechanisms underlying the formation of amyloid aggregates *in vivo* and in pathological conditions are still poorly understood. Post-translational modifications are known to affect protein structure and function and, recently, much attention has been devoted to the role played by non-enzymatic glycation in stimulating amyloid aggregation and cellular toxicity. In particular, glycation seems to have a determining role both in sporadic and familial forms of ALS and SOD1 has been shown to be glycated *in vivo* The aim of this study was to investigate the role of glycation on the amyloid aggregation process of both wild-type SOD1 and its ALS-related mutant G93A. To this aim, the glycation kinetics of both native and demetalated SOD have been followed using two different glycating agents, i.e., D-ribose and methylglyoxal. The effect of glycation on the structure and the amyloid aggregation propensity of native and ApoSOD has been also investigated using a combination of biophysical and biochemical techniques. In addition, the effect of SOD glycated species on cellular toxicity and reactive oxygen species (ROS) production has been evaluated in different cellular models. The results provided by this study contribute to clarify the role of glycation in amyloid aggregation and suggest a direct implication of glycation in the pathology of fALS.

## Introduction

Amyotrophic Lateral Sclerosis (ALS) is a neurological disease causing the death of motor neurons and muscular paralysis. Although, it is predominantly a sporadic disease, 10% of ALS cases are described as familial. Over 100 mutations, spread throughout the superoxide dismutase gene, are associated with familial ALS (fALS) (Rosen et al., 1993; Valentine et al., 2005; Andersen, 2006; Boillée et al., 2006; Chattopadhyay and Valentine, 2009). The presence of proteinaceous aggregates containing superoxide dismutase in motor neurons of patients and transgenic model mice suggests that the aggregation of this protein is directly related to the pathology of fALS. Eukaryotic copper, zinc superoxide dismutase SOD1 is a 32-kDa homodimeric metalloenzyme in which each of the two subunits forms an eight-stranded Greek key β-barrel and contains an active site that binds a catalytic copper ion and a structural zinc ion. *In vitro* studies have shown that wild type human SOD1, when lacking both its metal ions (ApoSOD), forms amyloid-like oligomers under physiological conditions of pH and temperature (Banci et al., 2007, 2008; Karch and Borchelt, 2008). Moreover, it has been reported that also some SOD1 mutants, many of them related to fALS (i.e., G93A) form soluble oligomeric species and that demetallation is the key factor for aggregation (Shaw and Valentine, 2007; Banci et al., 2008, 2009).

Amyloid aggregates and protein inclusions are a common pathological feature of many neurological disorders such as Huntington's, Alzheimer's and Parkinson's diseases. In these neurodegenerative diseases, misfolding, aggregation, and precipitation of proteins seem to be directly related to neurotoxicity. Amyloid fibrils share common structural features despite the considerable diversity in the primary sequence of the component proteins. In particular, they are typically composed of unbranched fibrils (about 10 nm in diameter) rich in β-sheet structures in which the ordered regions adopt a cross-β structure (Serpell, 2000; Fitzpatrick et al., 2013). Although, extensive studies performed on the aggregation process of several amyloidogenic proteins *in vitro* allowed the identification of many physiological factors involved, the molecular mechanisms underlying the formation of amyloid aggregates *in vivo* and in pathological conditions are still poorly understood. Post-translational modifications are known to affect protein structure and function; indeed some of them are known to affect proteins in detrimental ways and lead to their misfolding and accumulation. Reducing sugars play a key role in modifying proteins, forming advanced glycation end-products (AGEs) in a non-enzymatic process named glycation (Singh et al., 2001; Ulrich and Cerami, 2001). Protein glycation is an irreversible, non-enzymatic modification resulting from a chemical reaction between reducing sugars and primary amino groups in proteins (N-terminal, and arginine and lysine side chains). Glycation reaction produces very reactive intermediates that can promote the formation of intramolecular and intermolecular cross-links within AGE-modified protein monomers. All reducing sugars can promote glycation reactions and, between them, D-ribose is known to be the most active and its intracellular level can be quite high. D-glucose is the less reactive and its intracellular concentration is negligible, while dicarbonyl compounds, such as methylglyoxal and glyoxal, are far more reactive. Proteins in amyloid deposits are often found glycated suggesting a direct correlation between protein glycation and amyloidosis (Vitek et al., 1994; Münch et al., 1997; Kikuchi et al., 2000; Shults, 2006). In particular, glycation seems to have a determining role both in sporadic and familial forms of ALS; in fact spinal cord and brain samples were found to be glycated in patients (Chou et al., 1998, 1999). Moreover, SOD1 has been shown to be glycated *in vivo* and glycation sites have been identified (Arai et al., 1987). Further studies revealed that AGEs levels were high in patients carrying SOD1 mutations and in mutant SOD1 transgenic mice, while control cases did not display AGEs immunoreactivity (Kato et al., 2001; Takamiya et al., 2003).

Recently, much attention has been devoted to the role played by glycation in stimulating amyloid aggregation and cellular toxicity. Results obtained for different protein models indicate that, depending on the protein, glycation can both promote amyloid aggregation or induce formation of oligomeric species, stabilized by covalent cross-links AGE-derived, that do not evolve into amyloid aggregates (Bouma et al., 2003; Lee et al., 2009; Rondeau et al., 2010; Oliveira et al., 2011; Iannuzzi et al., 2013a, 2014, 2015; Adrover et al., 2014). Also, glycation can affect structural and physicochemical features of amyloid oligomers as well as their interaction to the cell membrane and subsequently modulate and/or induce the cell toxicity. However, AGEs modified proteins are always able to affect cell viability irrespective of amyloid properties. Indeed, the AGE-modified proteins are tightly involved in physiopathological cellular mechanisms through the interaction with specific cellular receptors (RAGE) that lead to the activation of signaling pathways involving inflammatory and apoptotic processes (Lue et al., 2005; Vicente Miranda and Outeiro, 2010).

The aim of this study was to investigate the role of glycation on the amyloid aggregation process of both wild-type SOD and its fALS-related mutant G93A. To this aim, we have followed the glycation kinetics of both native and demetalated SOD (for wild-type and G93A mutant) using two different glycating agents, i.e., D-ribose and methylglyoxal. Then, we have investigated the effect of glycation on the structure and the aggregation propensity of native and ApoSOD using a combination of biophysical and biochemical techniques. In addition, we have evaluated the effect of SOD glycated species on reactive oxygen species (ROS) production and cellular toxicity in different cellular models. The results provided by this study contribute to clarify the role of glycation in amyloid aggregation and suggest a direct implication of glycation in the pathology of fALS.

## Materials and methods

### Materials

Thioflavin T (ThT), 3-(4,5-dimethylthiazol-2-yl)-2,5-diphenyl-tetrazolium bromide (MTT), D-ribose, 2′,7′-dichlorofluorescin diacetate, methylglyoxal, (Sigma-Aldrich Co., St. Louis, MO). All other chemicals were of analytical grade. Methylglyoxal was further purified by distillation under low pressure and its concentration was determined spectrophotometrically using ε_284_ = 12.3 M^−1^cm^−1^ (Oya et al., 1999).

### Cloning

cDNA encoding the human intracellular CuZnSOD was purchased by OriGene (USA) and inserted in the pETDuet-1 vector (Novagen) by PCR using oligonucleotides that introduce *BamHI* site at the initiation codon and *NotI* site downstream of the stop codon. The gene encoding the yeast copper chaperone, yCCS, was isolated from Pichia pastoris GS115 genome and inserted in pETDuet-1 vector by PCR using oligonucleotides that introduce *NdeI* site at the initiation codon and *XhoI* site downstream of the stop codon. To enable coexpression of SOD gene and mutant variants with the yCCS in the same vector, the chaperone gene was inserted into the respective SOD plasmids.

The SOD_G93A mutant was constructed using a QuickChange mutagenesis kit (Stratagene). The identity of the mutation was confirmed by DNA sequencing and mass spectrometry of the purified proteins.

### Human SOD expression and purification

Recombinant human SOD (WT and G93A mutant) were co-expressed with yCCS in E. coli Rosetta-DE3 (Novagen). Cultures grown overnight on agar-plates containing 100 μg/ml ampicillin were used to inoculate liquid cultures of 2L LB-medium supplemented with 100 μg/ml ampicillin. The cultures were incubated at 37°C with shaking until OD_600_ reached 0.5. Expression of the proteins was induced by adding 1 mM isopropyl-β-d-thiogalactoside, CuSO_4_ (3 mM) and ZnSO_4_ (30 μM) were also added at the time of induction. The cultures were incubated at 20°C overnight and the cells were harvested by centrifugation (5000 g, 20 min). The resulting pellets were resuspended in 40 ml extraction buffer (50 mM phosphate, pH 8; 300 mM NaCl; 20 mM imidazole; 0.001 mg/ml DNase; 0.01 mg/ml RNase; 0.3 mM PMSF; half cocktail inhibitor tablet Complete (Roche) and lysed by ultrasonication. The lysates were cleared by centrifugation (15,000 *g*, 30 min).

SOD proteins were produced with a N-ter His-tag and purified by affinity chromatography using Ni-NTA agarose gel (Qiagen). The collected fractions were further purified by size exclusion chromatography on a Superdex 75 26/60 column (GE Healthcare) in 50 mM phosphate, pH 8, 150 mM NaCl (Figure S1). The metal content of the purified proteins was assessed by inductively coupled plasma atomic emission spectroscopy (ICP-AES) and measurements of enzymatic activity by the pyrogallol autoxidation method (Marklund and Marklund, 1974).

### ApoSOD preparation

Demetalated superoxide dismutase (ApoSOD) was obtained by treating the purified metalated SOD (Holo-SOD) with 50 mM EDTA at pH 3.0. After 10 min incubation at RT, the protein was loaded on a Superdex 75 26/60 column (GE Healthcare) in 50 mM phosphate, pH 8, 150 mM NaCl. This procedure allows fast refolding of the protein and getting rid of possible Holo-SOD still present. All buffers used for Apo-proteins were treated with Chelex 100 resin (Biorad) to remove trace metals. The apo state of the protein was verified by ICP-MS (Agilent Technologies, USA), and determined to contain a molar fraction of 0.03 Zn.

### Protein glycation and aggregation

Protein concentration was determined by absorbance (ε_280_ = 5500 M^−1^cm^−1^). Glycated SOD and ApoSOD were prepared mixing protein at a final concentration of 100 μM with 0.5 M D-ribose or 5 mM methylglyoxal in 50 mM NaH_2_PO_4_ buffer, 150 mM NaCl, pH 7.4, passed through a 0.22 μm filter and incubated at 37°C in sterile conditions. Protein in buffer without glycating agent was used as control. Also, a control sample having the same amount of glycating agent but without protein was incubated under identical conditions.

For aggregation studies, protein samples were incubated at 37°C under vigorous stirring with teflon balls, 1/8″ diameter (Polysciences, Inc.). Aliquots were collected in sterile conditions before analysis.

### Fluorescence measurements

Fluorescence measurements were performed on a Perkin Elmer Life Sciences LS 55 spectrofluorimeter. To assess the intrinsic fluorescence of AGEs (λ_ex_ 320 nm/λ_em_ 410 nm), glycated proteins at a final concentration of 8 μM were monitored at different incubation times with the glycating agents. The fluorescence intensity was corrected by subtracting the emission intensity of D-ribose/methylglyoxal solutions at different incubation times. ThT fluorescence (λ_ex_ 450/λ_em_ 482 nm) was monitored at different time intervals after addition of ThT to protein samples. Working concentrations were 8 μM for protein samples and 25 μM for ThT. As there is a partial overlap between ThT and AGEs emission spectra, the ThT fluorescence was corrected by subtracting the emission intensity of glycated samples before the addition of ThT.

### Circular dichroism (CD) measurements

CD spectra were recorded at 25°C on a JascoJ-715 spectropolarimeter using thermostated quartz cells of 0.1 cm. Spectral acquisition was taken at 0.2 nm intervals with a 4 s integration time and a bandwidth of 1.0 nm. An average of three scans was obtained for all spectra. Photomultiplier absorbance did not exceed 600 V in the spectral region analyzed. All measurements were performed under nitrogen flow and spectra were recorded after diluting six times the stock solution (final protein concentration 20 μM). Data were corrected for buffer contributions using the software provided by the manufacturer (System Software version 1.00) and transformed in mean residue ellipticity before analysis. Protein secondary structure estimation was performed using CDPro software, which contains three software packages, i.e., CDSSTR, CONTIN/LL, and SELCON3 (Sreerama and Woody, 2000).

### Gel electrophoresis (SDS-PAGE)

The glycation-induced oligomerization was monitored by the protein mobility shift on SDS-PAGE (15%) using Bio-Rad (USA) electrophoresis equipment. Ten micrograms of protein sample were loaded and the protein bands were stained with Coomassie Brilliant Blue.

### Transmission electronic microscopy (TEM)

Aliquots of protein samples (3 μL) were placed on the copper grid and allowed to dry. After 5–6 min uranyl acetate replacement stain 1X (3 μL) was loaded on the grid and air dried. Images were acquired using a Libra 120 (Zeiss) Transmission Electron Microscope equipped with Wide-angle Dual Speed CCD-Camera sharp:eye 2K (4Mpx.).

### Cell cultures and treatments

CPAE endothelial cells (ATCC# CCL-209) were cultured in Minimun Essential Medium (MEM) supplemented with 10% fetal bovine calf serum (USA Origin), 2.0 mM glutamine, 100 units/mL penicillin and 100 mg/mL streptomycin in a 5.0% CO_2_ humidified environment at 37°C. SH-SY5Y human neuroblastoma cells (ATCC# CRL-2266) were cultured in Eagle's Minimum Essential Medium (EMEM) supplemented with 10% fetal bovine serum.

For all experiments, cells in culture medium without protein and in the presence of non-glycated protein served as control. Before incubation with cells, protein glycated in the presence of 0.5 M D-ribose for 8 days was subjected to dialysis in sterile conditions to remove the free glycating agent.

### Cell viability assay

Cell viability was assessed as the inhibition of the ability of cells to reduce the metabolic dye 3-[4,5-dimethylthiazol-2-yl]-2,5-diphenyltetrazolium bromide (MTT) to a blue formazan product (Hansen et al., 1989; Sirangelo et al., 2009). CPAE and SH-SY5Y cells were seeded in 12-well plates at a density of 140,000 and 250,000 cells/well respectively. After indicated times of incubation with protein samples, cells were rinsed with phosphate buffer solution (PBS). A stock solution of MTT (5 mg/mL in PBS) was diluted 10 times in cell medium and incubated with cells for 3 h at 37°C. After removing the medium, cells were treated with isopropylalcohol, 0.1 M HCl for 20 min. Levels of reduced MTT were assayed by measuring the difference in absorbance at 570 and 690 nm. Data are expressed, as average percentage reduction of MTT with respect to the control ±S.D. Data are an average from five independent experiments carried out in triplicate.

### Detection of intracellular ROS

Intracellular ROS were detected by means of an oxidation-sensitive fluorescent probe 2′,7′-dichlorofluorescin diacetate (DCFH-DA). EC were grown in a six-well plates, pre-incubated with DCFH-DA for 30 min and then incubated with protein samples for 6 h. Control experiments were performed using untreated cells. After incubation, cells were washed twice with PBS buffer and then lysed with Tris-HCl 0.5M, pH 7.6, 1% SDS. The non-fluorescent DCFH-DA is converted, by oxidation, to the fluorescent molecule 2′,7′-dichlorofluorescein (DCF). DCF fluorescence intensity was quantified on a Perkin Elmer Life Sciences LS 55 spectrofluorimeter using an excitation wavelength of 488 nm and an emission wavelength of 530 nm. Data are expressed as average ±S.D. from five independent experiments carried out in triplicate.

### Statistical analysis

For statistical analysis, we used a two-tailed Student's *t*-test with unequal variance at a significance level of 5% unless otherwise indicated.

## Results and discussion

### Glycation of human SOD and ApoSOD promotes structural changes

**Figure 1:**
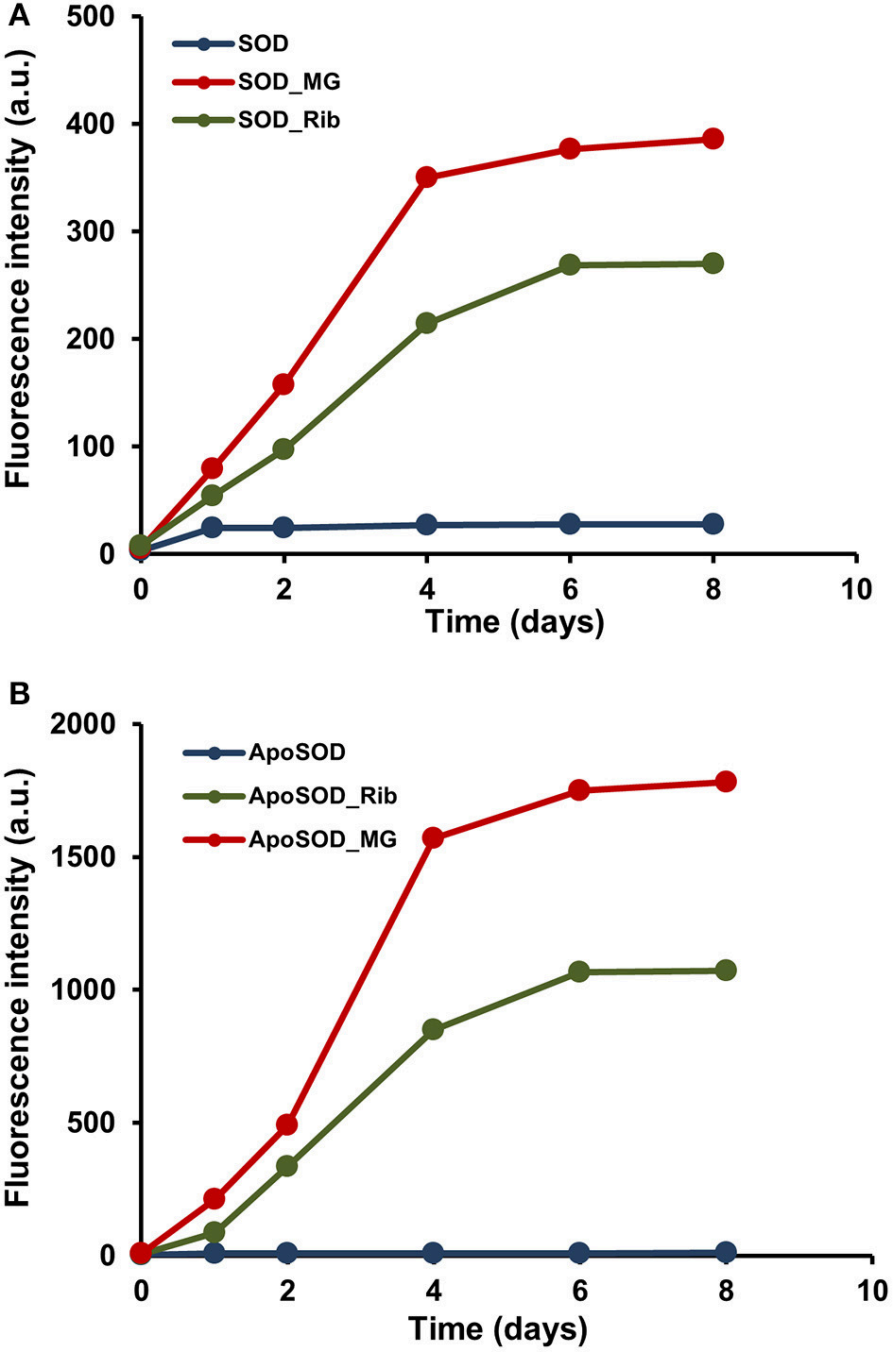
**Glycation kinetics of human SOD and ApoSOD**. Protein glycation monitored by fluorescence spectroscopy for human SOD **(A)** and ApoSOD **(B)**. Proteins were incubated in the absence (blue) and in the presence of 0.5 M D-ribose (green) or 5 mM methylglyoxal (red) and samples analyzed by emission fluorescence (λ_ex_ 320/λ_em_ 410 nm) at different time points. Protein concentration was 8 μM, other experimental details are described in the Materials and Methods section.

To check whether native SOD and ApoSOD can be glycated *in vitro*, the proteins were incubated at 37°C in the presence of D-ribose or methylglyoxal alternatively and samples were analyzed at different time points (days) by fluorescence spectroscopy. Indeed, glycation of a protein results in the appearance of a new fluorescence derivative centered at 410 nm (λ_ex_ = 320 nm) that is widely used to monitor the AGEs formation (Matiacevich and Buera, 2006). Figure 1 shows the time course of the emission intensity at 410 nm for SOD **(A)** and ApoSOD **(B)** incubated with 0.5 M D-ribose and 5 mM methylglyoxal. In both proteins, the fluorescence emission intensity increased markedly with incubation time indicating that both native SOD and ApoSOD can be glycated *in vitro* in the presence of D-ribose or methylglyoxal. In particular, methylglyoxal was much more effective than D-ribose in affecting the AGEs formation. Human SOD and ApoSOD alone, used as a negative control, showed no fluorescence at 410 nm.

**Figure 2:**
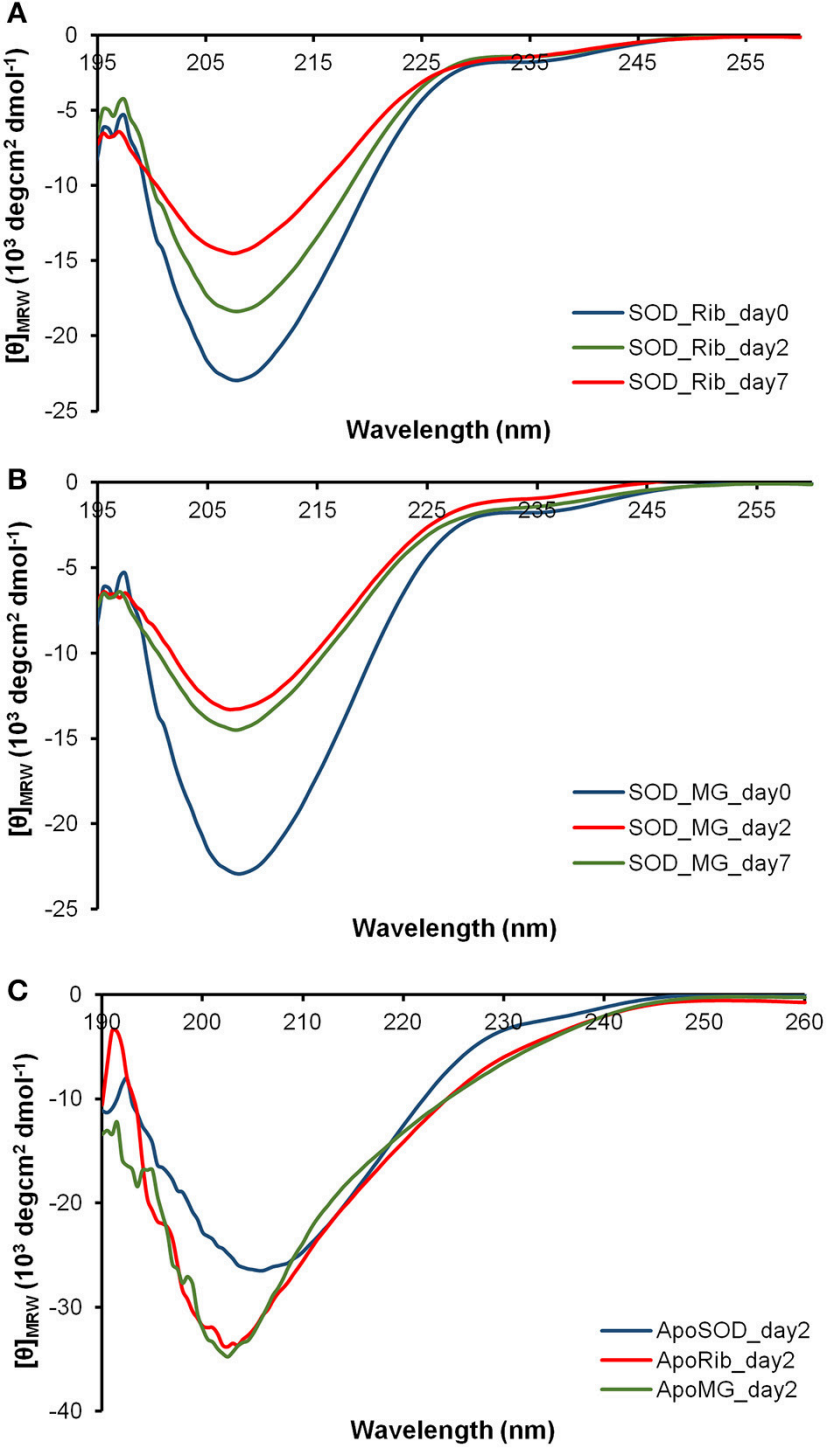
**Effect of glycation on the secondary structure of human SOD and ApoSOD**. Time dependence of the far-UV CD activity of the human SOD in the presence of 0.5 M D-ribose **(A)** or 5 mM methylglyoxal **(B)** at indicated time points. In **(C)** are shown far-UV CD spectra recorded at 2 days of incubation for the ApoSOD in the absence (blue) and in the presence of 0.5 M D-ribose (red) or 5 mM methylglyoxal (green). Experimental details are described in the Materials and Methods section.

**Figure 3:**
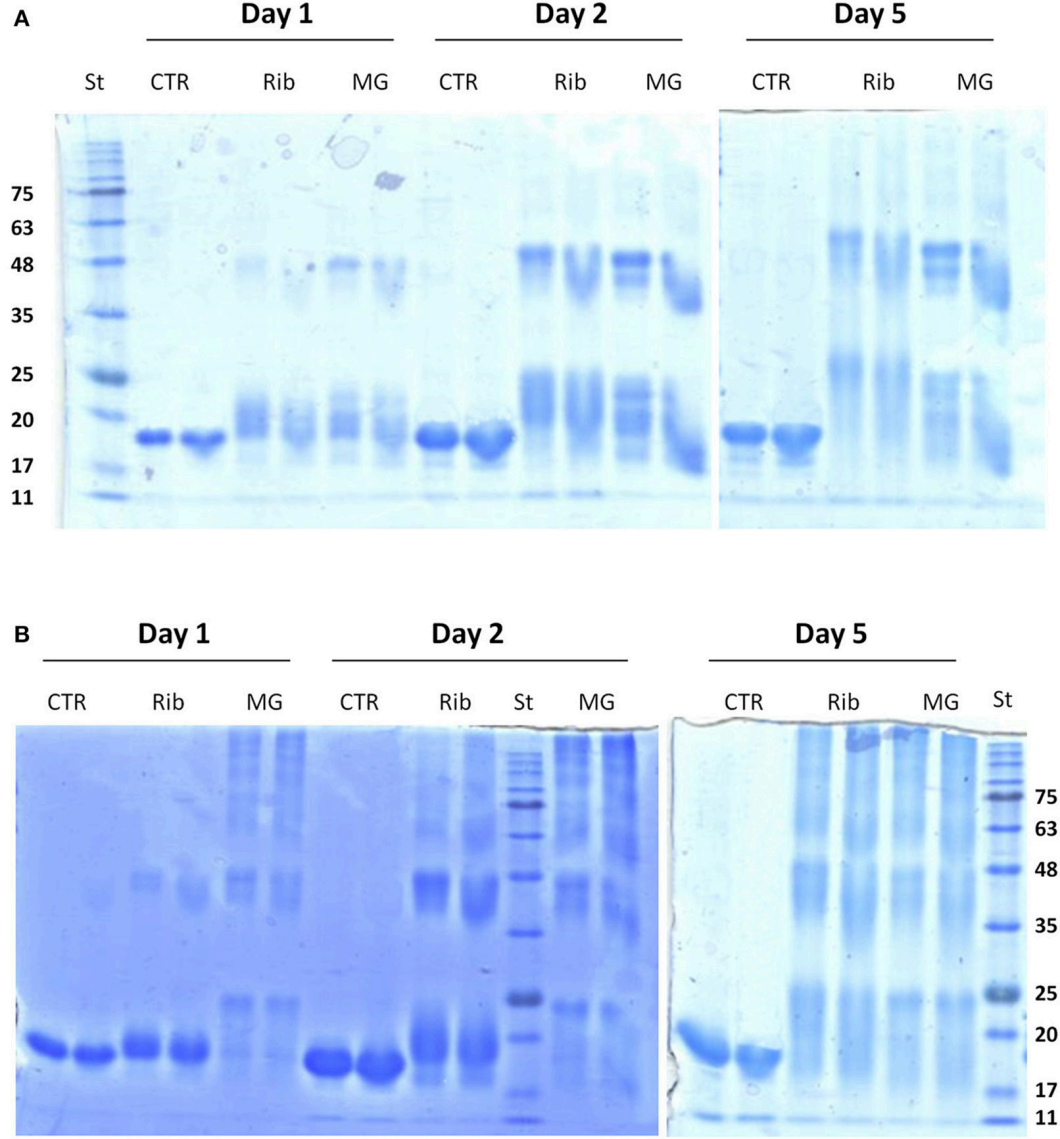
**Effect of glycation on the oligomerization of human SOD and ApoSOD**. SDS-PAGE analysis of human SOD **(A)** and ApoSOD **(B)** incubated in the absence and in the presence of 0.5M D-ribose or 5 mM methylglyoxal. Aliquots were taken at 1, 2, and 5 days of incubation at 37°C. At each time point two different protein concentration were analyzed: 100 μM (left lane) and 200 μM (right lane). Ten micrograms of protein sample were loaded for each lane. Other experimental details are described in the Materials and Methods section.

The dichroic activity in the far-UV region (far-UV CD) was monitored in human SOD and ApoSOD upon glycation in order to detect changes in the secondary structure induced by glycation. The proteins were incubated at 37°C in the presence and in the absence of 0.5M D-ribose or 5 mM methylglyoxal and the samples were analyzed by CD spectroscopy at different time points (Figures 2, 3).

In order to follow changes in the secondary structure induced by glycation in human SOD and ApoSOD we monitored the dichroic activity in the far-UV region (far-UV CD) upon glycation. After incubation of each protein at 37°C in the presence and in the absence of 0.5M D-ribose or 5 mM methylglyoxal, samples were analyzed by CD spectroscopy at different time points (Figures 2, 3). As expected, no difference in the CD activity was detected for the proteins without glycating agent at different incubation times (data not shown). Spectra of human SOD recorded at different times of incubation with D-ribose or methylglyoxal are shown in Figures 2A,B respectively. In both cases, at the beginning of the glycation reaction, the spectrum exhibited a strong minimum at 208 nm indicative of a predominantly beta structure conformation. Although, CD spectra of glycated and non-glycated SOD do not exhibit strong shape variation compared to the non-glycated protein, possess a very similar shape, the reduction in the dichroic activity at 208 nm observed as glycation proceeds is indicative of indicate suggests loss of beta secondary structure upon glycation. No further variations in the dichroic activity were observed at longer times (monitored for 30 days) and no as well as no light scattering effects was detected, indicative of suggesting absence of protein aggregation. Deconvolution analysis of the CD spectra confirmed the identified a loss of beta structure in human SOD upon glycation and a corresponding increase of unordered fraction (Table 1). Indeed, at the beginning of the glycation reaction, the protein possess mostly beta (34%) and unordered structure (41%). As the reaction proceeds, the protein loses beta-structure (around 50% after 7 days of reaction) and gains mostly unordered structure. The results show that Also, there is a strong correspondence between the estimated content of secondary structure and the AGEs formation kinetics reported in Figure 1 clearly indicating that the reduction in the helical content beta structure is directly related to the glycation extent. Such structural alterations induced by glycation could be responsible for the loss of activity recently observed for native SOD glycated *in vitro* in the presence of glucose, glyoxal, and methylglyoxal (Jabeen et al., 2007; Khan et al., 2014).

**Table 1:** **Secondary structure content of human SOD in the absence and in the presence of D-ribose or methylglyoxal at different incubation times**.

	**SOD**	**SOD/MG**			**SOD/Rib**		
		**Day0**	**Day2**	**Day7**	**Day0**	**Day2**	**Day7**
α-helix	0.05	0.05	0.04	0.04	0.05	0.04	0.03
β-strand	0.34	0.34	0.20	0.18	0.34	0.27	0.19
turn	0.20	0.20	0.15	0.15	0.20	0.18	0.15
unordered	0.41	0.41	0.61	0.63	0.41	0.51	0.63

*Analysis was performed as described in the Materials and Methods section and results are expressed as percentage*.

Different results were obtained for demetalated ApoSOD (Figure 2C). As expected, this protein, compared to the native SOD, showed a lower extent of secondary structure. Indeed, loss of metal binding is known to induce partial unfolding of the protein and loss of activity in human SOD (Banci et al., 2007, 2008; Chattopadhyay and Valentine, 2009). In Figure 2C are reported the far-UV CD spectra recorded at the beginning of the glycation process, and after 2 days of glycation in the presence of D-ribose or methylglyoxal. No further variations in the dichroic activity were observed at longer times (monitored for 30 days) and no light scattering was detected, indicative of absence of protein aggregation. These data indicate that glycation both with D-ribose or methylglyoxal promotes fast unfolding in ApoSOD. Also, the loss of secondary structure is much stronger compared to that observed in the native protein indicating that the ApoSOD is much more sensitive to glycation compared to the native SOD. Deconvolution analysis of the CD spectra confirmed the loss of beta structure upon glycation and a corresponding increase of unordered fraction. In Table 2 is reported the secondary structure content for ApoSOD at different glycation times.

**Table 2:** **Secondary structure content of human ApoSOD in the absence and in the presence of D-ribose or methylglyoxal at different incubation times**.

	**ApoSOD**	**ApoSOD/MG**		**ApoSOD/Rib**	
		**Day0**	**Day2**	**Day0**	**Day2**
α-helix	0.02	0.02	0.01	0.02	0.01
β-strand	0.28	0.28	0.08	0.28	0.08
turn	0.15	0.15	0.10	0.15	0.10
unordered	0.55	0.55	0.81	0.55	0.81

*Analysis was performed as described in the Materials and Methods section and results are expressed as percentage*.

### Glycation of human SOD and ApoSOD and oligomerization

Glycation has been indicated as a contributory factor in the formation of high molecular weight protein species which originate from inter molecular cross-links among AGEs adducts (Lee et al., 2009; Oliveira et al., 2011; Iannuzzi et al., 2014). In order to monitor the increase in the apparent molecular weight of glycated adducts, glycated species of human SOD and ApoSOD were analyzed by SDS-PAGE in reducing conditions (Figure 3). Samples of SOD **(A)** and ApoSOD **(B)** were analyzed at different incubation times (1, 2, and 5 days) in the absence and in the presence of 0.5 M D-ribose or 5 mM methylglyoxal at two different molar ratio. Results shown in Figure 3A indicate that glycation in native SOD results in an increase in the molecular weight already at 1 day of incubation with both glycating agents. Such increase can be ascribed to the addition of sugar molecules to the protein. Increase of glycation extent and stabilization of SOD dimers were observed at longer incubation times. No higher order oligomers were observed at any time both with D-ribose or methylglyoxal. These data indicate that glycation in native SOD does not promote oligomerization but only covalent dimer stabilization.

Differently, ApoSOD incubated with both D-ribose or methylglyoxal clearly showed the appearance of high molecular weight bands in time thus indicating that glycation promotes the formation of covalent crosslinked oligomeric species (Figure 3B). The process seems to be much faster in the presence of methylglyoxal compared to D-ribose. Taken together, these results suggest that the glycation reaction promotes oligomerization only in ApoSOD suggesting that protein glycation could be a triggering factor in the aggregation of ApoSOD associated to fALS.

### Glycation inhibits amyloid fibrils formation in ApoSOD

**Figure 4:**
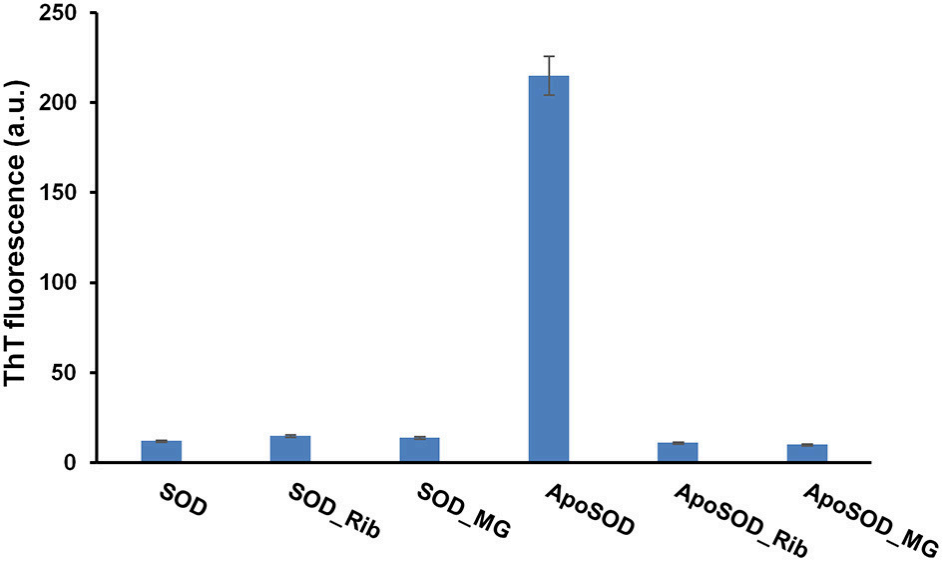
**Effect of glycation on the amyloid aggregation of human SOD and ApoSOD**. Human SOD and ApoSOD glycated in the presence of 0.5 M D-ribose or 5 mM methylglyoxal for 8 days were incubated under stirring at 37°C and analyzed at 10 days of incubation by ThT fluorescence assay. Similarly, samples incubated in the absence of glycating agent were also tested. ThT fluorescence emission was recorded at 482 nm upon excitation at 450 nm. Other experimental conditions are described in the Materials and Methods section.

Amyloid fibrils formation generally involves a series of stages including aggregation of soluble oligomers through unspecific interactions, formation of protofibrillar structures and eventually their assembly into mature fibrils through a nucleation and elongation process (Lee et al., 2007; Fitzpatrick et al., 2013; Iannuzzi et al., 2013b; Knowles et al., 2014). Generally, oligomeric intermediates are the most toxic species compared to the mature fibrils (Malmo et al., 2006; Iannuzzi et al., 2007; Vilasi et al., 2008; Stefani, 2012; Sirangelo et al., 2014). ApoSOD is known to form amyloid aggregates in native conditions at 37°C upon perturbation (stirring) (Banci et al., 2007, 2008). In order to study the effect of glycation on the aggregation process of the human SOD, we tested the ability of glycated SOD and ApoSOD to form amyloid aggregates in native conditions upon stirring. To this aim, human SOD and ApoSOD glycated by D-ribose or methylglyoxal for 8 days were incubated at 37°C upon stirring and samples were analyzed at different times of incubation (up to 60 days) by ThT fluorescence assay. ThT fluorescence is a widely used method for detecting amyloid formation as ThT specifically binds amyloid structures exhibiting a strong fluorescence increase (LeVine, 1993). In Figure 4 is reported the ThT fluorescence intensity at 10 days of incubation in aggregation conditions. As expected, the non-glycated ApoSOD is able to bind ThT thus indicating the formation of amyloid species upon stirring. Interestingly, no ThT fluorescence increase was detected for the glycated sample clearly indicating that glycation inhibits amyloid formation in ApoSOD. Similar results were obtained for glycated samples at longer incubation time (up to 60 days). Differently to apoSOD, the native SOD is unable to form amyloid aggregates upon stirring at 37°C (Banci et al., 2007, 2008). As expected, no ThT fluorescence increase was observed for native SOD in aggregating conditions. Interestingly, also the glycated samples did not show ThT fluorescence thus indicating that glycation does not promote amyloid formation in native SOD.

**Figure 5:**
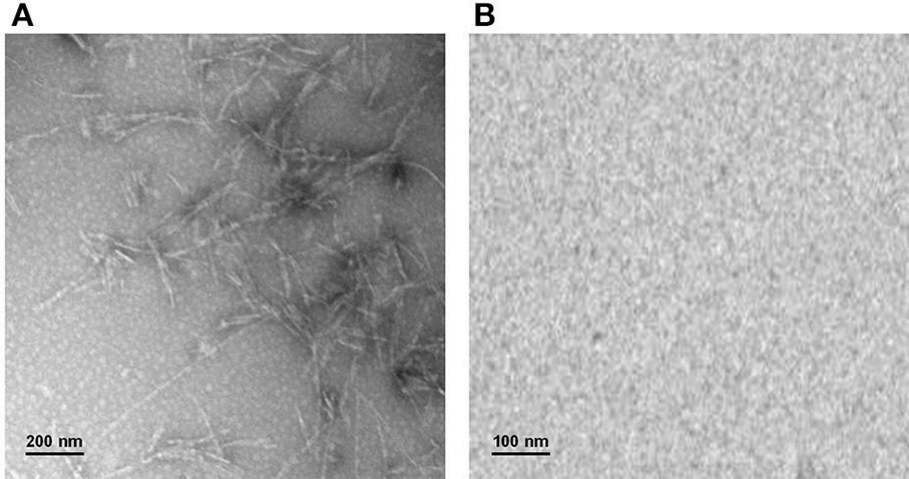
**Transmission electron microscopy**. ApoSOD glycated in the absence **(A)** and in the presence **(B)** of 0.5 M D-ribose for 8 days was incubated under stirring at 37°C and analyzed at 10 days of incubation by TEM microscopy. Experimental details are described in the Materials and Methods section.

Taken together, these results strongly suggest that glycation with both D-ribose or methylglyoxal strongly inhibits amyloid fibrils formation in ApoSOD and does not promote amyloid aggregation in native SOD. Transmission electron microscopy (TEM) measurements further confirmed these data (Figure 5). Indeed, consistent with the ThT staining, the TEM images recorded for ApoSOD at 10 days of incubation in aggregation conditions revealed the presence of mature fibrils only in the absence of glycating agent. No prefibrillar aggregates and/or mature fibrils were detected in the sample glycated with D-ribose. Similar results were obtained in the presence of methylglyoxal.

### Cytotoxicity of glycated SOD and ApoSOD

**Figure 6:**
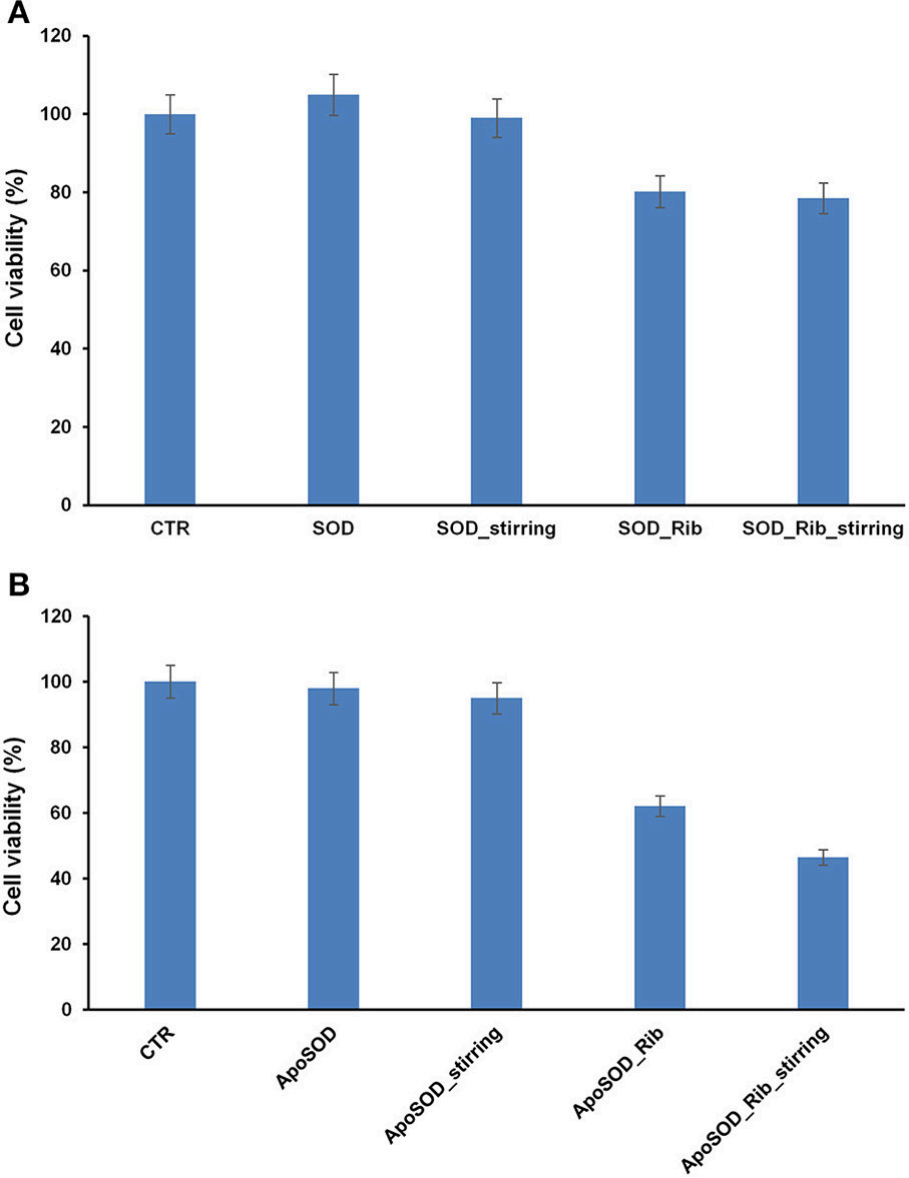
**Effect of glycated SOD and ApoSOD on cell viability**. SH-SY5Y cells were exposed for 24 h to different SOD **(A)** and ApoSOD samples **(B)** and cell viability was evaluated by MTT assay. Protein incubated in the absence (SOD/ApoSOD) and in the presence (SOD_Rib/ApoSOD_Rib) of D-ribose for 8 days. These samples were also tested after 10 days in aggregation conditions (_stirring). CTR represents untreated cells. Data are expressed as average percentage of MTT reduction ±SD relative to control cells from triplicate wells from 5 separate experiments (*p* < 0.01). Protein concentration was 20 μM, other experimental conditions are described in the Materials and Methods section.

As oligomeric intermediates are the most toxic species respect to mature fibrils, to better clarify the role of glycation in amyloid aggregation, viability of cells exposed to ribose-glycated SOD and ApoSOD before and after 10 days of aggregating conditions (stirring at 37°C) was also evaluated (Figure 6). The cytotoxicity of such glycated protein was studied in both endothelial (CPAE) and neurotypical (SHSY5Y) cell lines and very similar results were obtained for both cellular models.

As expected, no toxicity was detected for the native SOD both before and after aggregating conditions. Interestingly, a weak toxicity was shown for glycated forms. In particular, the same extent of toxicity was observed for glycated SOD both before and after stirring thus indicating that toxicity is not related to the formation of cytotoxic oligomers but only to glycated products (Figure 6A).

When the cells were incubated with ApoSOD no cytotoxicity was shown as expected. Similarly, the ApoSOD incubated for 10 days upon stirring did not alter cell viability indicating that amyloid fibrils formed upon stirring are harmless. On the contrary, the cell viability decreased significantly when cells were exposed to the glycated ApoSOD (Figure 6B). In particular, glycated ApoSOD was able to induce strong cytotoxicity both before and after stirring. The strong MTT reduction (around 40%) observed in cells exposed to the glycated proteins suggests that glycation promotes the formation of highly cytotoxic species AGEs-associated, although inhibiting amyloid aggregation in ApoSOD. These results suggest that the non-amyloidogenic oligomers are the species responsible for cytotoxicity.

Indeed, AGEs modified proteins are known to affect cell viability through the interaction with specific cellular receptors (RAGE) that lead to the activation of signaling pathways involving inflammatory and apoptotic processes and irrespectively of their amyloid properties. (Lue et al., 2005; Vicente Miranda and Outeiro, 2010). Also, the higher toxicity observed for the glycated ApoSOD compared to the native SOD could be associated to its higher degree of glycation.

**Figure 7:**
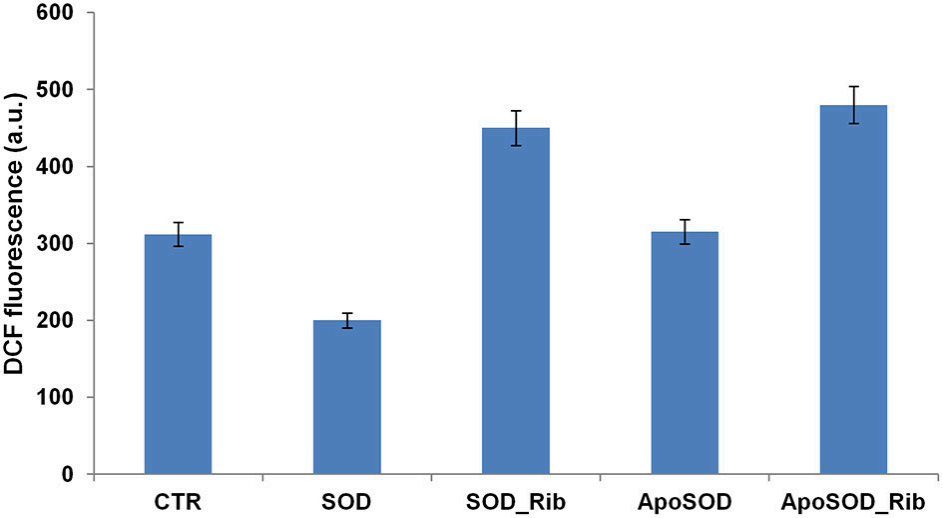
**Effect of glycated SOD and ApoSOD on ROS production**. ROS levels measured by the DCFH-DA fluorescence assay as described in Material and Methods section. SH-SY5Y cells were exposed for 6 h to SOD and ApoSOD previously incubated in the presence and in the absence of D-ribose for 8 days. CTR represents untreated cells. Protein concentration was 20 μM, other experimental conditions are described in the Materials and Methods section.

Moreover, to test whether oxidative stress plays an important role in the cell death associated to glycated SOD and ApoSOD, we measured the intracellular ROS levels using the redox-sensitive fluorescent dye DCFH-DA (Figure 7). Cell exposed to the native SOD showed reduced production of intracellular ROS compared to control cells, probably due to the intrinsic antioxidant activity of the protein, while cell exposed to ApoSOD were not. This might be due to the loss of activity in the demetalated protein. Interestingly, both glycated SOD and ApoSOD were able to trigger intracellular ROS production. This effect might be associated to the loss of activity in the native SOD upon glycation and to the ability of AGEs products to induce oxidative stress (Jabeen et al., 2007; Wei et al., 2009; Kong et al., 2011; Khan et al., 2014). This observation is in line with previous reports showing that ROS production increases in AGEs-induced toxicity for several cell lines (Yamagishi and Takeuchi, 2004; Khan et al., 2013; Iannuzzi et al., 2016). An important issue dealing with the signaling cascade evoked by AGE treatment is the mechanism by which it causes oxidative stress. Previous studies indicate that AGE proteins prepared *in vitro* possess similar cross-reactive AGE epitopes that are common to proteins modified by AGEs *in vivo* and that interaction of these molecules with RAGE is associated with ROS generation (Kislinger et al., 1999; Han et al., 2011, 2014). Thus, the ROS production observed in cells exposed to glycated SOD and ApoSOD could be associated to signaling pathways activated by the RAGE-AGE interaction. Further studies will be needed to better clarify the molecular mechanisms underlying the cytotoxicity induced by glycated SOD and ApoSOD.

### Human G39A_SOD glycation and oligomerization

**Figure 8:**
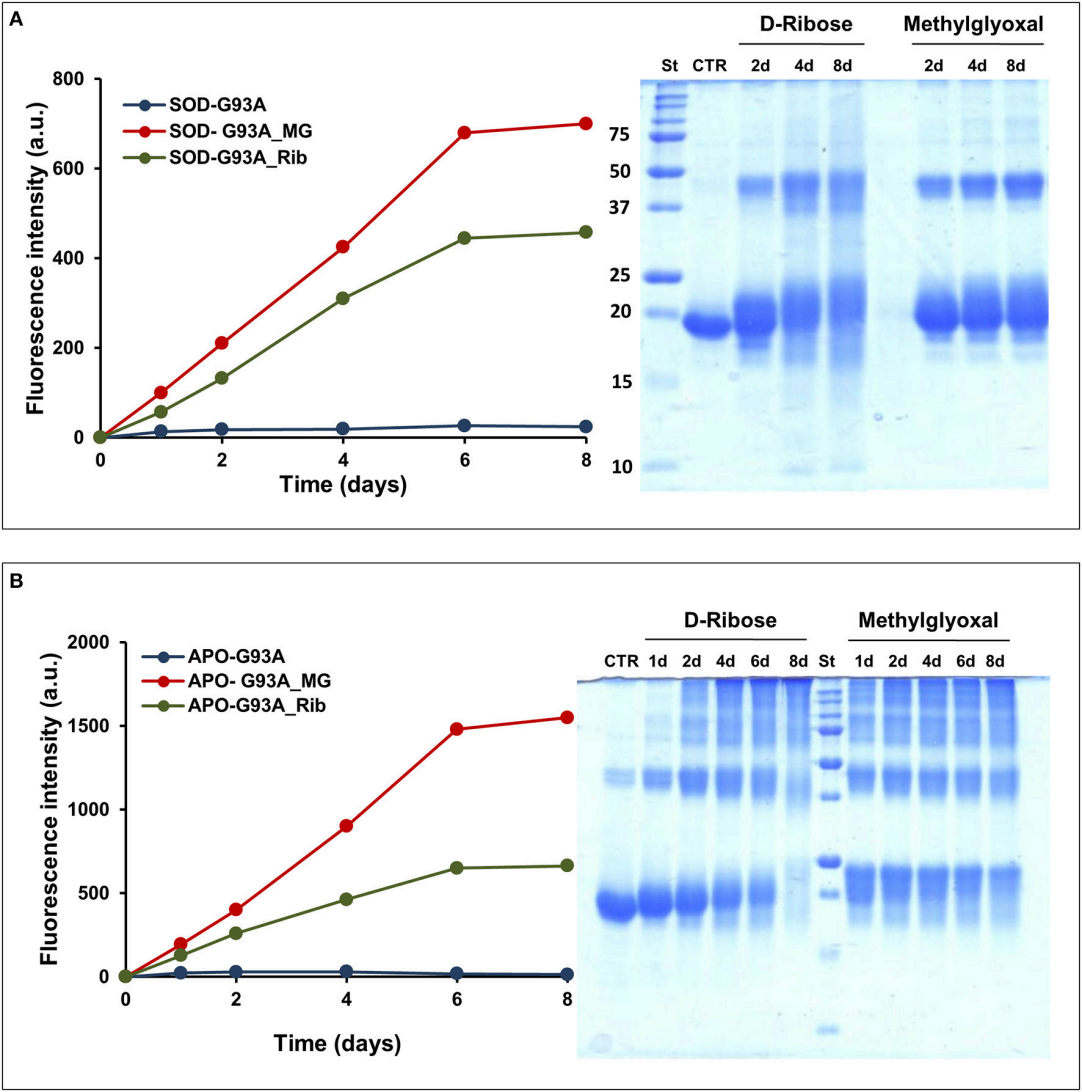
**Glycation kinetics and oligomerization of G93A_SOD and G93A_ApoSOD**. Glycation kinetics and oligomerization monitored by fluorescence spectroscopy (λ_ex_ 320 nm/λ_em_ 410 nm) and SDS-PAGE for G93A_SOD **(A)** and G93A_ApoSOD **(B)**. Proteins were incubated in the absence (blue) and in the presence of 0.5 M D-ribose (green) or 5 mM methylglyoxal (red) and samples were analyzed at different time points. Other experimental details are described in the Materials and Methods section.

The effect of glycation was also monitored for the fALS-related mutant SOD_G93A. The G93A is one of the most severe mutation associated to fALS and it has been reported to strongly destabilize the apo-state of the protein leading to fast formation of amyloid fibrils in physiological conditions (Shaw and Valentine, 2007; Banci et al., 2008, 2009; Karch et al., 2009; Luchinat et al., 2014). Figure 8 shows the glycation kinetics recorded for the G93A_SOD **(A)** and ApoG93A_SOD **(B)** by AGEs fluorescence and SDS-PAGE analysis. The proteins were incubated in the presence of 0.5 M D-ribose or 5 mM methylglyoxal and samples analyzed at different time points. As for the wild-type protein, the glycation extent is much higher in the Apo-G93A compared to the G93A protein thus suggesting a higher exposure of glycation sites in the demetalated form. Also, comparing these results with those obtained for the wild-type protein (Figure 1), the relative AGEs fluorescence intensities are much higher in the mutant thus suggesting that the G93A_SOD mutant is much more susceptible to glycation respect to the wild-type protein. These results are in perfect agreement with data obtained *in vivo* by Takamiya and coworkers showing a higher degree of glycation in the G93A_SOD mutant compared to the wild type protein. In this paper they also suggest that the higher susceptibility of mutated SOD to glycation *in vivo* is an important factor in the pathogenesis of fALS (Takamiya et al., 2003).

Glycated samples analyzed by SDS-PAGE show that glycation with both D-ribose or methylglyoxal promotes covalent dimerization in the G93A_SOD and high oligomerization in the ApoG93A_SOD. Also in this respect, the effect of glycation on protein oligomerization of G93A_SOD is similar to that observed for the wild-type protein.

In addition, we tested the ability of glycated G93A_SOD and ApoG93A_SOD to form amyloid aggregates at physiological conditions of pH and temperature under stirring. The non-glycated proteins were used for comparison. As for the wild-type protein, both the G93A_SOD and its glycated form were unable to form amyloid aggregates as suggested by the absence of ThT fluorescence (data not shown). These data indicate that, as for the wild-type protein, glycation does not promote amyloid formation in the G93A_SOD mutant.

**Figure 9:**
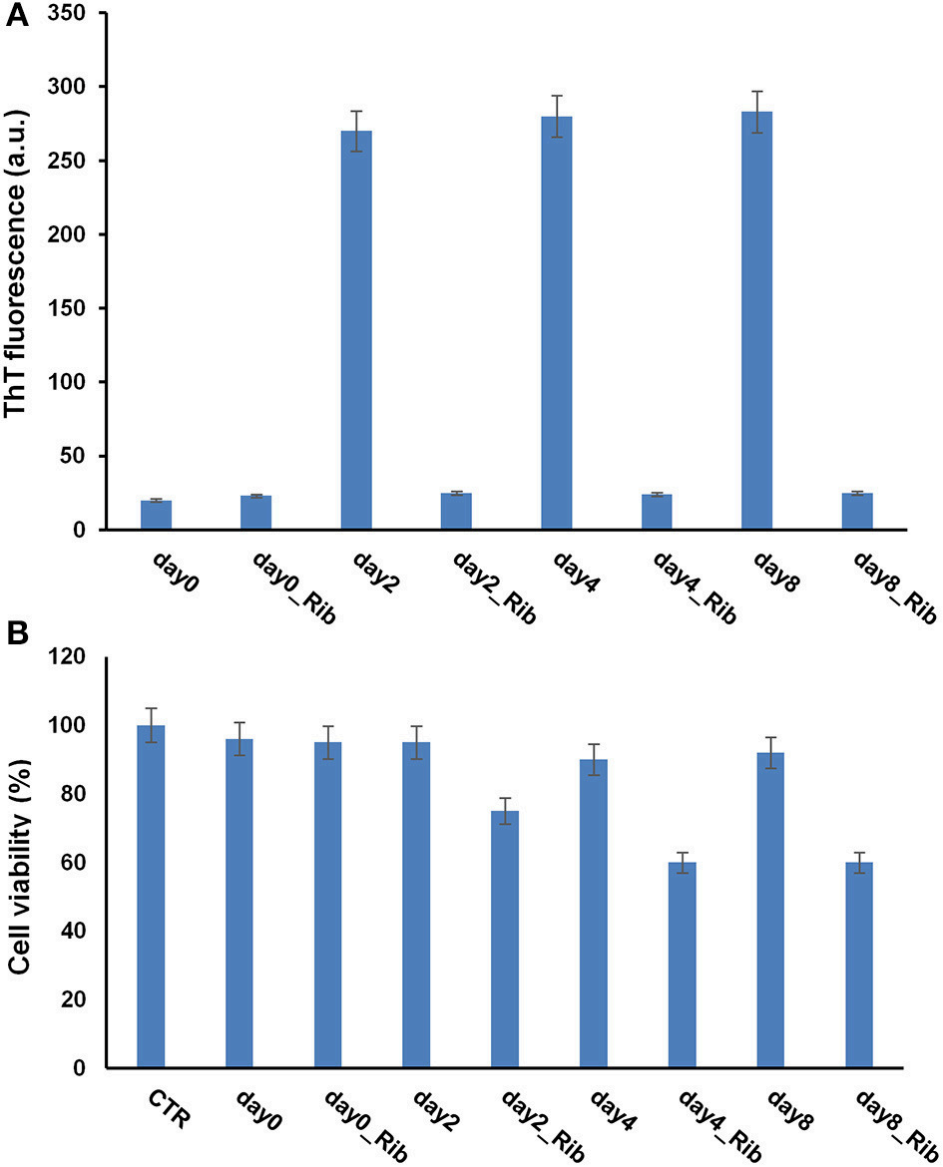
**Effect of glycation on the amyloid aggregation and cytotoxicity of G93A_ApoSOD**. G93A_ApoSOD glycated in the presence and in the absence of D-ribose for 8 days were incubated under stirring at 37°C and analyzed at different time points (days) by ThT fluorescence assay **(A)**. The same samples were tested for cytotoxicity by the MTT assay **(B)**. CTR represents untreated cells. Data are expressed as average percentage of MTT reduction ±SD relative to control cells from triplicate wells from 5 separate experiments (*p* < 0.01). Other experimental conditions are described in the Materials and Methods section.

Different results were obtained for the Apo G93A_SOD. As expected, this protein has a strong propensity to form amyloid aggregates compared to the wild-type protein, as indicated by an early appearance of ThT fluorescence at 2 days in aggregating condition (Figure 9A). Differently, ThT fluorescence increase was detected for the wild type Apo protein at 8–10 days of incubation. Such higher aggregation propensity could be associated to a strong destabilization of the apo-structure in the mutant, as suggested by far-UV CD spectroscopy (Figure S2). Interestingly, as observed for the wild type ApoSOD, no ThT fluorescence was observed for glycated ApoG93A_SOD in time, thus indicating that glycation inhibits amyloid aggregation also in the amyloidogenic mutant. Moreover, the same samples analyzed for ThT fluorescence were tested for cytotoxicity by MTT assay (Figure 9B). No toxicity was observed for the non-glycated ApoG93A_SOD at any aggregation time being in the harmless fibrillar state. Differently, the glycated species altered the cell viability in a time-dependent manner related to the AGE extent as observed for the wild-type protein. These data suggest that glycation, even in the strongly amyloidogenic ApoG93A_SOD mutant, is able to inhibit amyloid aggregation also at early stage of the aggregation process. However, the glycated species show an intrinsic toxicity related to the AGEs formation.

## Conclusions

The present study clearly shows that both native SOD and ApoSOD can be efficiently glycated *in vitro* by D-ribose or methylglyoxal and glycation seems to induce protein unfolding and loss of secondary structure. As observed for other model proteins, the effect of glycation on amyloid aggregation may not be generalized as strongly depending on the protein structure. Indeed, protein glycation, being a post-translational modification, can differently affect the aggregation process in promoting, accelerating and/or stabilizing on-pathway and off-pathway species.

Results obtained on native SOD indicate that glycation does not promote amyloid aggregation in this protein. At the same time, glycation strongly inhibits amyloid aggregation in the amyloidogenic Apo-state of the protein both for the wild type and for the G93A fALS-related mutant. In addition, AGEs-modified SOD and ApoSOD were shown to induce cell toxicity and oxidative stress in different cellular models which may be likely mediated by the interaction of AGE-modified proteins with RAGE. Further studies will be needed to better clarify the molecular mechanisms underlying the cytotoxicity induced by the AGE-modified SOD.

In conclusion, these data suggest that glycation is unlikely to be a triggering factor in SOD amyloid aggregation *in vivo*. However, the cytotoxicity induced by SOD glycated species suggests that glycation could directly interfere with fALS pathogenesis through the signaling pathways activated by the AGEs species.

## Author contributions

Conceived and designed the experiments, CI, FV, IS, GM, and GI; Performed the experiments, FV, CI; Analyzed the data, FV, CI, and IS; Contributed reagents/materials/analysis tools, CI, IS, and GM; Wrote the paper, CI.

## Funding

This work was supported by a grant from MIUR (Finanziamento per Rientro dei Cervelli “Rita Levi Montalcini”), and “Regione Campania (L.R. N.5- 28.03.2002)”. The funders had no role in study design, data collection and analysis, the decision to publish, or preparation of the manuscript.

### Conflict of interest statement

The authors declare that the research was conducted in the absence of any commercial or financial relationships that could be construed as a potential conflict of interest.
