# Supplementary material for: Glycation in Demetalated Superoxide Dismutase 1 Prevents Amyloid Aggregation and Produces Cytotoxic Ages Adducts
Source: Front Mol Biosci. 2016 Sep 16;3:55. doi: 10.3389/fmolb.2016.00055 (PMC5026054; doi:10.3389/fmolb.2016.00055)
Supplement: Supplementary file 1 [file Image1.PDF]

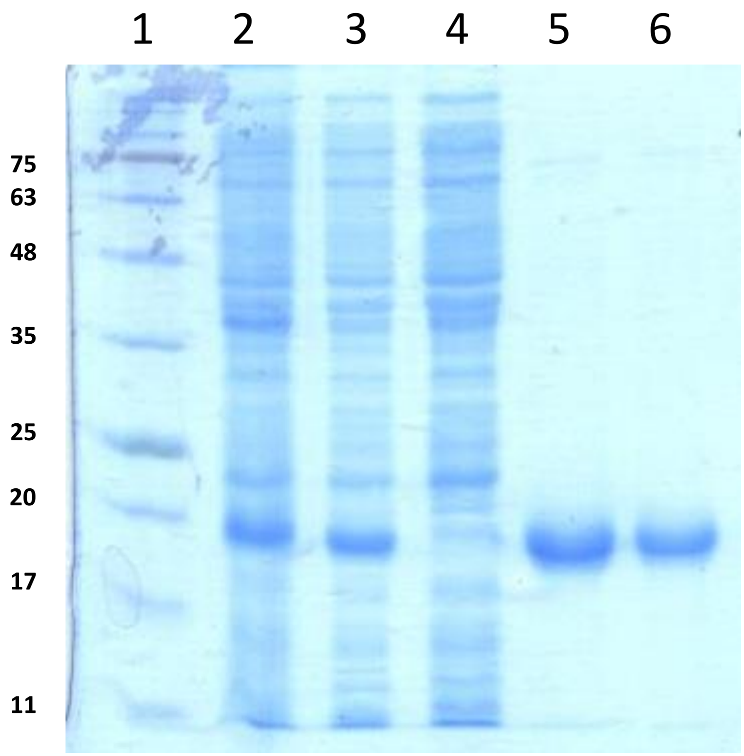

Figure S1: Purification of human SOD.

Coomassie-stained SDS-PAGE gel showing protein profiles of: lanes 1, molecular mass protein ladder; 2 whole cell extract; 3, supernatant after cell disruption and centrifugation; 4, fraction excluded by the Ni-NTA agarose gel; 5, pooled active fractions from Ni-NTA affinity chromatography; 6, protein after size exclusion chromatography.
