# Supplementary figures and images for: Glycation in Demetalated Superoxide Dismutase 1 Prevents Amyloid Aggregation and Produces Cytotoxic Ages Adducts
Source: Front Mol Biosci. 2016 Sep 16;3:55. doi: 10.3389/fmolb.2016.00055 (PMC5026054; doi:10.3389/fmolb.2016.00055)

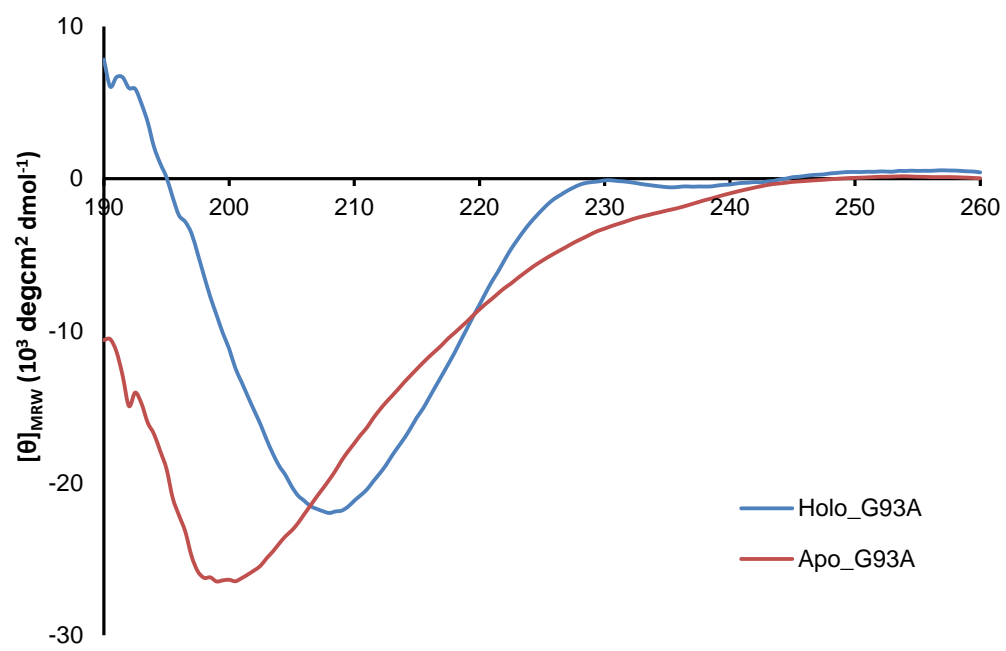

**Figure S2: Far-UV CD spectra of G93A\_SOD and ApoG93A\_SOD**

Supplement: Supplementary file 2 [file Image2.PDF]
